# The Assessment of the Usefulness of Prenatal Magnetic Resonance Imaging in the Diagnosis of Central Nervous System Defects

**DOI:** 10.3390/diagnostics11091723

**Published:** 2021-09-20

**Authors:** Magdalena Kołak, Izabela Herman-Sucharska, Małgorzata Radoń-Pokracka, Małgorzata Stolarek, Anna Horbaczewska, Hubert Huras

**Affiliations:** 1 Department of Obstetrics and Perinatology, Medical College, Jagiellonian University, 23 Kopernika Str., 31-501 Krakow, Poland; malgorzata.radon@gmail.com (M.R.-P.); hubert.huras@uj.edu.pl (H.H.); 2 Department of Radiology, Medical College, Jagiellonian University, 19 Kopernika Str., 31-501 Krakow, Poland; isuchar@poczta.onet.pl; 3 Faculty of Medical Sciences and Health Sciences, Kazimierz Pułaski University of Technology and Humanities in Radom, 29 Malczewskiego Str., 26-600 Radom, Poland; malgorzata.stolarek@wp.pl; 4 Department of Endocrynological Gynecology, Medical College, Jagiellonian University, 23 Kopernika Str., 31-501 Krakow, Poland; anna.horbaczewska@yahoo.pl

**Keywords:** magnetic resonance, central nervous system, prenatal, diagnosis

## Abstract

Central nervous system (CNS) abnormalities cause about 40% of infant deaths in the first year of life. In case of the detection of abnormalities by ultrasound, a pregnant woman should be offered prenatal magnetic resonance imaging (pMRI). The aims of our study were: (1) to evaluate the effectiveness of pMRI in the diagnosis of selected fetal CNS defects; and (2) to assess the possibility of replacing postnatal tests with prenatal magnetic resonance. The prospective and observational study was conducted between 2014 and 2017 at the University Hospital in Krakow. Patients with suspected CNS defects of the fetus were qualified for pMRI in the third trimester of pregnancy. Sixty patients were included in the study group. Prenatal MRI was characterized by low accuracy in the diagnosis of complex brain defects. Cohen’s kappa coefficient κ = 0.21 (95% CI 0.00–0.46). No evidence was found suggesting the replacement of postnatal tests with pMRI. MRI was characterized by low consistency of diagnoses in the case of complex brain defects. The possibility of replacing postnatal studies with pMRI was not supported.

## 1. Introduction

Congenital malformations occur in 2–4% of newborns. Approximately 7000 children annually are born with a developmental disorder. The incidence of central nervous system (CNS) defects is estimated at 6.7/10,000 live births [1]. CNS abnormalities are considered the most serious malformations. They cause about 40% of infant deaths in the first year of life [2].

In the case of prenatal suspicion of fetal defect, postnatal verification is necessary. In the case of birth of a child with a CNS defect, in the first days of life a cranial ultrasound (US) examination is conducted to confirm the diagnosis. Then, in the first months of life, the infant is subjected to more detailed imaging—magnetic resonance imaging (MRI) or computed tomography (CT). These techniques are characterized by much greater accuracy in comparison with ultrasound examination. However, their accessibility and cost-efficiency is markedly lower. In addition, the examination duration is longer and the involvement of a larger group of medical personnel is required. Another consideration is that the examinations must be performed, at times, under general anesthesia. This subjects the baby to the additional burdens of intubation and the administration of anesthetic drugs. Prenatal MRI is also not free of bias. Fetal movements may affect results of the imaging. The picture of some defects may differ depending on the gestational age.

The first goal of the study was to evaluate the effectiveness of prenatal magnetic resonance imaging (pMRI) in the diagnosis of fetal defects of the central nervous system. The second goal of the study was to assess the possibility of replacing postnatal tests with pMRI.

## 2. Materials and Methods

The prospective and observational study was conducted between 2014 and 2017 at the Obstetrics and Perinatology Department, Jagiellonian University Hospital in Krakow, in association with the Neonatology Department, the Jagiellonian University Hospital in Krakow, the Jagiellonian University Children’s Hospital in Krakow, and at the Obstetrics and Gynecology Hospital “Ujastek” in Krakow. The study was granted approval by the Bioethics Committee of Jagiellonian University no. KBET/157/B/2014 from 26 June 2014.

### 2.1. The Study Group

The Obstetrics and Perinatology Department of the Jagiellonian University Hospital in Krakow is a tertiary hospital; all patients with high-risk pregnancy, including those with fetal malformations, are referred to the Pregnancy Pathology Clinic for further diagnosis and treatment if necessary. The fetal ultrasound examinations were performed by specialists in the field of neurosonography. The study was performed using a GE Voluson ultrasound system (General Electric Healthcare, Zipf, Austria) with hybrid transducers with frequencies between 4–8 MHz. The ultrasound examination was performed in accordance with the guidelines of the International Society of Ultrasound in Obstetrics and Gynecology, the Polish Ultrasound Society and the recommendations of the Ultrasound Division of the Polish Gynecological Society.

### 2.2. Criteria for Inclusion in the Study

(1) Suspicion of a CNS defect in the fetus;

(2) Finding fetal abnormalities that may be associated with a CNS defect (cardiac rhabdomyoma, arthrogryposis);

(3) A positive family history that may be associated with a CNS defect (tuberous sclerosis in the father of the child or in a child from a previous pregnancy).

### 2.3. Exclusion Criteria

(1) Presence of lethal fetal defects;

(2) Claustrophobia of the patient;

(3) Metal implants in the patient’s body;

(4) pMRI not performed in third trimester of pregnancy (before 28 w).

### 2.4. Study Protocol

The patients who met the inclusion criteria underwent pMRI in the third trimester of pregnancy, optimally in the late third trimester (around 35 w). After delivery, the newborns had their diagnoses verified by US, MR, or CT examination.

### 2.5. Prenatal MRI

Prenatal MRI was performed in the third trimester of pregnancy, between 27–38 weeks of pregnancy, in the Magnetic Resonance Center of the Obstetrics and Gynecology Hospital “Ujastek” in Krakow and in the “Voxel” Center at the University Children’s Hospital in Krakow. Patients underwent imaging after eating a light meal. Before the test, no sedative agents were administered. The patients were lying in the supine position with their right side slightly raised to avoid inferior vena cava syndrome. Prenatal MRI scans were performed on a Siemens Magnetom Espree 1.5 Tesla MRI scanner (Siemens Healthcare, Erlangen, Germany). Gradients were greater than 20 mT/s and the rate of change of field strength was greater than 100 T/ms. The study protocol included T2- and T1-weighted sequences, true-FISP, dark-fluid, and DW-EPI. Scans were obtained routinely in three orthogonal planes over the relevant anatomical region. T2-W fast sequence for brain imaging was Half-fourier Acquisition Single-shot Turbo spin Echo (HASTE) (TR 1200 ms, TE 168 ms, slice thickness 3–4 mm, FOV 270 × 270 mm, matrix 256 × 192, NEX 2). T1-W imaging with fat suppression (FS) was acquired during breath-holding (TR 120 ms, TE min full, slice thickness 4–5 mm, FOV 400 × 320 mm, matrix 320 x 224, NEX 1). Breath-hold Gradient-Echo (General Electric Healthcare, Zipf, Austria) sequences with the bSSFP (True-FISP) (TR 3 ms, TE 1 ms, slice thickness 4–5 mm, FOV 400 × 300 mm, matrix 256 × 144) were employed in all cases of excessive fetal motion, causing artifacts for the great vessels and heart imaging. Diffusion-Weighted (DW) Echo-Planar Imaging (EPI) (TR 5300 ms, TE 79 ms, slice thickness 4–5 mm, NEX 4, FOV 360 × 360, matrix 256 × 192, flip angle 90°, TI 185; b0, b50 and b500 s/mm^2^) with the application of gradients oriented in three planes was used to study the kidneys, lungs, brain, and placenta.

### 2.6. Verification of Results

The final postnatal diagnoses were based on medical records, clinical examination, and imaging results (ultrasound and MRI) provided by the University Hospital Neonatology Clinic in Krakow, the University Children’s Hospital in Krakow, and the Neonatology Department of the Obstetrics and Gynecology Hospital “Ujastek” in Krakow. Ultrasound of the newborn was performed using a GE Voluson ultrasound system (General Electric Healthcare, Zipf, Austria), with sector or linear transducers with a frequency between 7.5–8 MHz. Ultrasound examinations were performed in accordance with the applicable guidelines. MRI scan of the newborn was performed using the Siemens Magnetom Espree 1.5 T system (Siemens Healthcare, Erlangen, Germany). T1 and T2-dependent sequences were used. The layer thickness was from 4 to 5 mm, and the interval between successive layers from 1 to 5 mm. Imaging was performed in transverse, sagittal, and frontal planes.

### 2.7. Statistical Analysis of Results

The Shapiro-Wilk test was used to assess the normality of the quantitative variables’ distribution. Depending on the normality of the distribution, continuous variables are presented as mean ± standard deviation or as medians and quartiles. The U-Mann-Whitney test was used to examine the differences between two independent groups. Multidimensional logistic regression was also applied. Qualitative variables are presented as percentages. In order to analyze the differences between two qualitative variables, the Chi2 test was used. The Cohen’s kappa coefficient was calculated. Statistical analyses were performed using the STATISTICA 12 software (StatSoft, Tulsa, Oklahoma, United States). The value of α = 0.05 was assumed as the level of significance. Detailed statistical analysis in relation to certain CNS defect is presented in Table 1.

### 2.8. Definitions

Ventriculomegaly: depending on the degree of ventricular expansion, it is divided into mild (10–12 mm), moderate (12–15 mm), or severe (over 15 mm) [3].

Complex CNS defect: two or more coexisting defects of CNS.

## 3. Results

**Figure 1:**
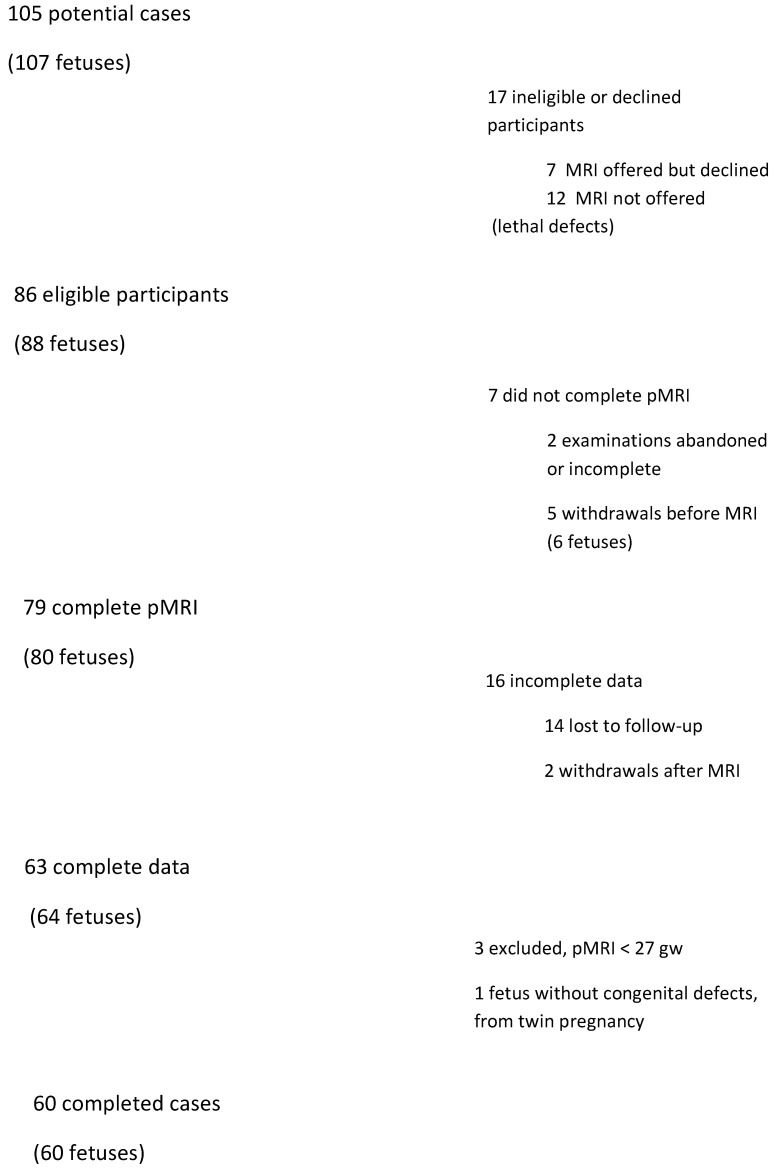
Flow chart of cases included in the study.

During the study, 105 patients received care at the Pregnancy Pathology Clinic due to suspected fetal CNS defects or obstetric history of CNS defects. In twelve cases, lethal defects were found and these patients were excluded from the study. Ten of these patients decided to terminate the pregnancy. In total, 60 patients were included in the study group: 54 cases due to suspected CNS defects, 5 cases due to other defects and 1 case due to a family history of tuberous sclerosis. Detailed information on the selection of the study group is shown in Figure 1.

### 3.1. Study Sample Features

The mean age of pregnant women subjected to pMRI was 28 ± 6 years, and they ranged from 16 to 43 years old. The majority of patients in the study group were in their first pregnancy (58%). Patients in their second and third pregnancy accounted for 12 (20%) and 11 (18.33%), respectively. In the study group, six (10%) patients had spontaneous abortion in their medical history. In the study group, 59 patients (98.3%) were single pregnancies. In one case, a dichorionic twin pregnancy was complicated by CNS defect of one of the fetuses. In nine (15%) patients, the pregnancy was additionally complicated by other diseases, including chronic hypertension, hypothyroidism, gestational diabetes, and CMV infection.

In 15 (25%) cases, one or more complications of pregnancy occurred, including congenital heart disease (ventricular septal defect (VSD), atrioventricular septal defect (AVSD), coarctation of the aortic arch, cardiomegaly, atrial septal aneurysm, cardiac rhabdomyoma, urinary system defects (hydronephrosis), limb defects, fetal growth disorders, oligohydramnios, polyhydramnios, arthrogryposis, and abnormal karyotype.

The median gestational age was 35 w (33–36 w).

### 3.2. Consistency of Diagnosis

#### 3.2.1. Ventriculomegaly

**Figure 2:**
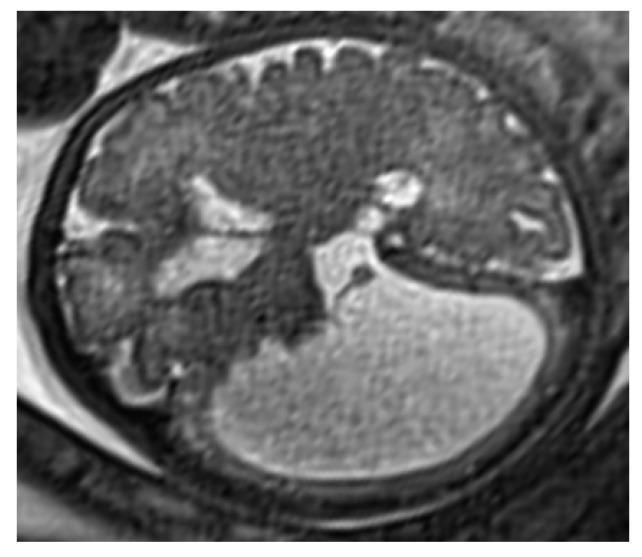
MRI picture of ventriculomegaly.

Isolated ventriculomegaly was suspected in 30 (50%) fetuses. In seven (11.67%) cases it was a suspicion of an isolated defect; however, in two newborns, additional abnormalities were detected in the postnatal examination. In one case, cerebellar hypoplasia was found, while in the other, white matter injury. In three (5%) cases, the abnormality was overdiagnosed. However, in one case, no fetal abnormality was found. MRI picture of ventriculomegaly is shown in Figure 2.

#### 3.2.2. Central Line Defects

##### Agenesis of Corpus Callosum

Twenty (33.33%) cases of suspected corpus callosum agenesis were analyzed. There were 14 (23.33%) consistent cases, of which 13 were diagnosed with complete agenesis of corpus callosum (cACC), and one partial (pACC). In three (5%) cases, the discrepancies concerned the diagnosis of cACC vs. pACC. Agenesis of corpus callosum was overdiagnosed in three (5%) fetuses (two partial, one complete).

In four cases, pACC was not diagnosed in pMRI, and additional abnormalities were found in one of the false-negative neonates: brain gray matter heterotopy and delayed myelination of brain white matter.

##### Holoprosencephaly

The analysis examined one case of syntelencephaly, a brain defect that is a form of holoprosencephaly characterized by an abnormal midline connection of the cerebral hemispheres between the posterior frontal and parietal regions. The comparison of the results was consistent, although additional features of brain demyelination were found in the postnatal study.

##### Agenesis of the Cavum Septum Pellucidum

**Figure 3:**
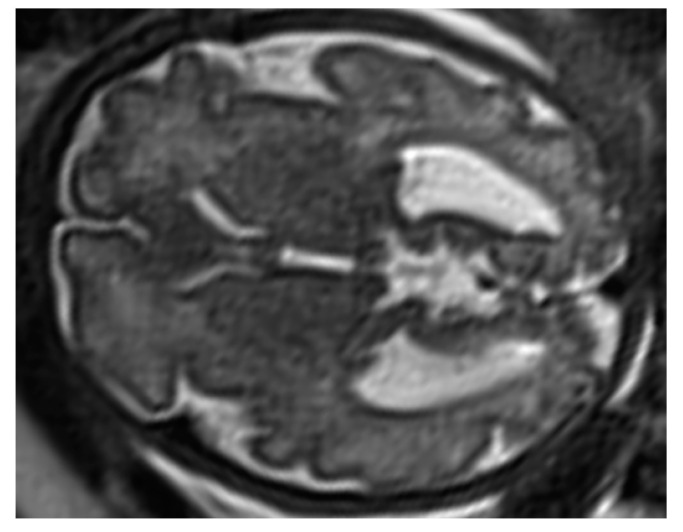
MRI picture of CVS defect.

The isolated absence of cavum septum pellucidum (CSP) was found in three (5%) cases. The absence of CSP was confirmed in all of them, and the presence of an optic junction was confirmed, excluding septo-optic dysplasia. In one case, the discrepancy in the results concerned whether the cavum agenesis was partial or complete. In one fetus (1.67%), the defect coexisted with ventriculomegaly, and in this case there was also a discrepancy of whether the cavum agenesis was partial or complete. For midline defects, the McNemara test χ^2^ 0.0 (*p* = 1.0) was used. Cohen’s kappa coefficient κ = 0.55 (95% CI 0.34–0.77). CSP defect in MRI is shown in Figure 3.

#### 3.2.3. Defects of the Posterior Fossa

##### Defects of the Cerebellum

**Figure 4:**
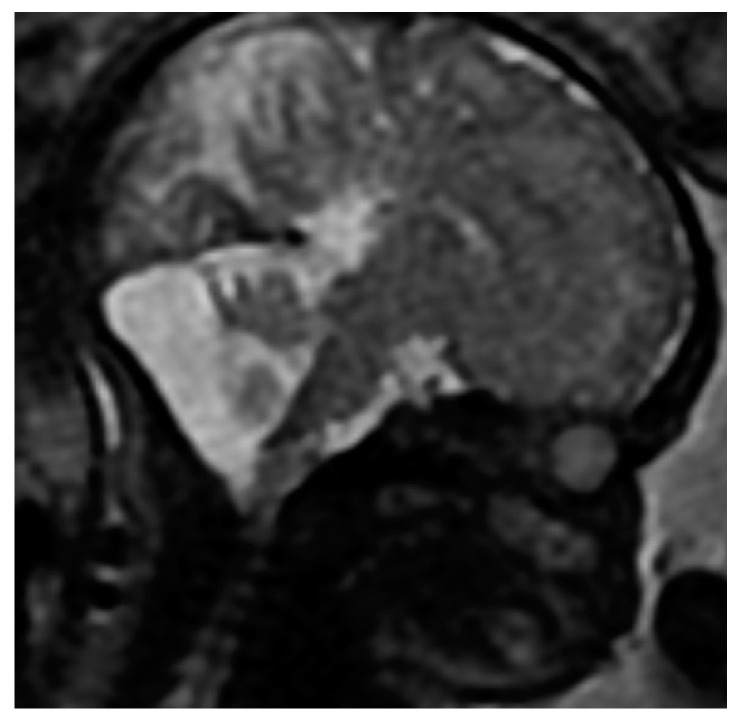
MRI picture of vermis dysgenesis.

In five (8.3%) cases, a cerebellar defect was suspected, and in three (5%) cases there was no compatibility between the pMRI and the postnatal examination. In two (3.33%) cases, prenatally, the cerebellum was classified as hypoplastic or dysgenetic, while after birth the normal cerebellum was found. In the last case (1.67%), cerebellar vermis dysgenesis was suspected prenatally and Dandy-Walker syndrome was diagnosed postnatally (Figure 4).

In one case, cerebellar hypoplasia was not diagnosed prenatally.

##### Dandy-Walker Syndrome

Dandy-Walker syndrome was suspected in three (5%) fetuses. In two (3.33%) cases, the diagnoses were consistent, but in one (1.67%) fetus no coexisting complex brain defect was diagnosed. In one (1.67%), the difference concerned the cerebellar vermis–prenatal agenesis was suspected, and postnatal hypoplasia was found. In one (1.67%) child, as mentioned in the cerebellar abnormalities section, cerebellar vermis dysgenesis was prenatally suspected and Dandy-Walker syndrome was diagnosed postnatally.

##### Arnold-Chiari Syndrome

Arnold-Chiari syndrome was suspected in four (6.67%) cases, and consistent in three (5%) cases. In one (1.67%), postnatal syndrome Arnold-Chiari was excluded. In defects of the posterior cranial fossa, the McNemara test χ^2^ 0.57 (*p* = 0.45) was used. Cohen’s kappa coefficient κ = 0.6 (95% CI 0.33–0.86).

#### 3.2.4. Disorders of Neural Migration and Proliferation

##### Lysencephaly

**Figure 5:**
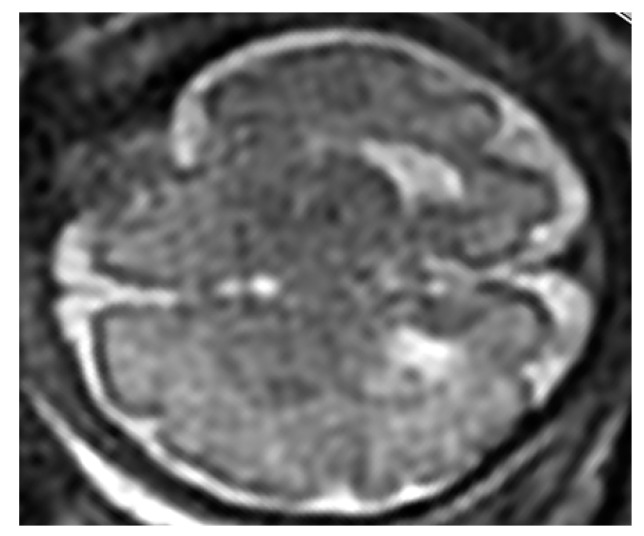
MRI picture of lissencephaly.

**Figure 6:**
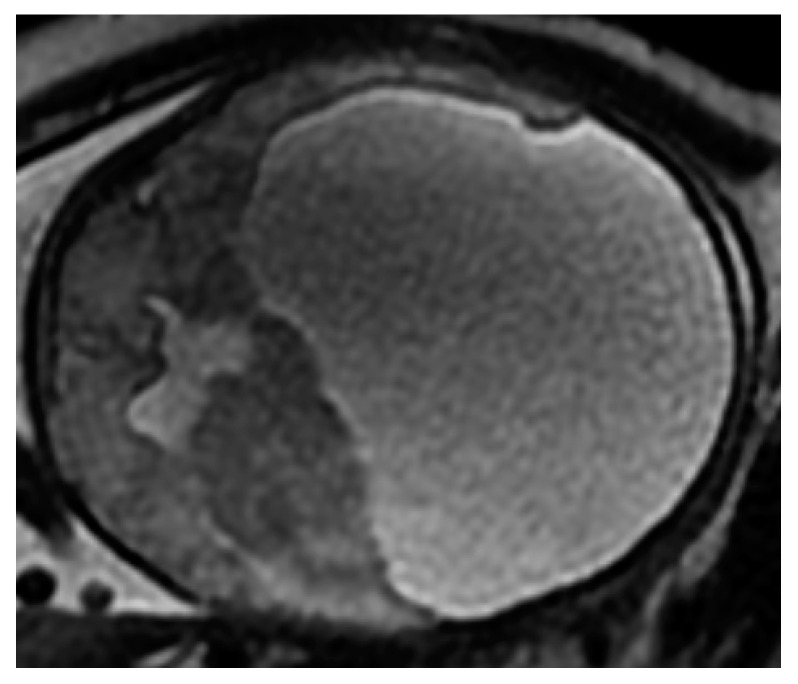
MRI picture of hydrancephaly.

Two fetuses (3.33%) were prenatally suspected of having lissencephaly (pMRI performed at 35 w in both cases). The first case was consistent (Figure 5), and in the second case, folding disorders were overdiagnosed. In one (1.67%) fetus, lissencephaly was not detected. The pregnancy was complicated by hydranencephaly, pMRI performed at 36 w (Figure 6). The prenatal MRI indicated that the folding was difficult to assess. Postnatal diagnosis was completed after insertion of the ventrico-peritoneal valve and decompression of the ventricular system.

##### Schizencephaly

There were two (3.33%) cases of schizencephaly. They were consistent in both pre- and postnatal findings (pMRI performed at 34 w in the first case and at 36 w in the second).

##### Microcephaly

The abnormality was suspected in two (3.33%) fetuses. In both, the underlying diagnosis was consistent. In the second case, in pMRI, agenesis of the corpus callosum was overdiagnosed. In six (10%) cases, pMRI did not reveal comorbid disorders: delayed myelination of white matter (three cases, 5%), gray matter heterotopia (two cases, 3.33%), periventricular white matter damage. pMRI performed at 36 and 37 w. In cases of neural migration and proliferation disorders, the McNemara χ^2^ 2.29 test (*p* = 0.13) was used. Cohen’s kappa coefficient κ = 0.53 (95% CI 0.23–0.83).

#### 3.2.5. Other Irregularities

##### Abnormalities Related to Peripheral Nervous System Defects

Twelve (20%) cases were analyzed, and indications for magnetic resonance were: fetal rhabdomyoma and family history of tuberous sclerosis—five (8.33%) cases in total. Further indications were: observation of the posterior cranial fossa (one case, 1.67%), narrowing of the skull in the frontal region (one case, 1.67%), intraventricular haemorrhage (one case, 1.67%), shortening of the limbs (one case, 1.67%), arthrogryposis (two cases, 3.33%), and suspected CMV infection (one case, 1.67%). In 10 (16.67%) cases, diagnoses were consistent and normal development of the brain was confirmed. The normal picture of the brain was found in the fetus suspected of having intraventricular bleeding. In the last child, brain calcifications were suspected prenatally, which were not confirmed by postnatal examination.

##### Meningo-Spinal Hernia

Three cases (5%) were analyzed, all with coexisting Chiarii II malformation. The pre- and postnatal results were consistent.

##### Arachnoid Cyst

Five (8.33%) cases were analyzed. In two (3.33%), the pre- and postnatal diagnoses were similar. Among the discrepancies, the following were observed: in the first one, a cyst was overdiagnosed, and in the second, a cyst was omitted and the pMRI was performed due to suspicion of cerebellar dysgenesis. In the latter case, the Dandy-Walker variant was diagnosed postnatally. For other abnormalities, the McNemara test χ2 0.13 (*p* = 0.72) was used. Cohen’s kappa coefficient κ = 0.64 (95% CI 0.42–0.87). Finally, the consistency of pre and post-natal diagnoses as a complete clinical picture was analyzed. In the case of complex CNS defects and the detection of an inconsistency in diagnosis in one of the main categories (midline defects, posterior fossa defects, migration disorder, ventriculomegaly), the overall diagnosis was considered inconsistent. In 18 (30%) cases, complex brain defects were found (midline defect + posterior fossa defect; midline defect + migration disorder; ventriculomegaly + midline defect; posterior fossa defect + migration disorder; ventriculomegaly + posterior fossa defect). Cohen’s kappa coefficient κ = 0.21 (95% CI 0.00–0.46).

#### 3.2.6. Additional Irregularities Detected in pMRI

Additional irregularities were detected in pMRI. These included: interthalamic adhesion, ocular irregularities, hypotelorism, and disorders of the tear ducts. Defects of other systems were also detected: urinary tract disorders including obstructive uropathy, vesicoureteral reflux, a duplex collecting system; cystic lung disease; and focal changes in the liver.

## 4. Discussion

### 4.1. Main Findings

The study included 60 female patients with fetuses suspected of a CNS defect, who, from June 30, 2014 to June 30, 2017, received care at the Obstetrics and Perinatology Clinic of Jagiellonian University Collegium Medicum. The study group is representative and proportional to cohorts studied in other centers [1,2,4]. This was an observational study. Its assumptions were as close as possible to the course of clinical practice. Thanks to this, the results can be used in everyday life. Not every newborn with a CNS defect needs an MRI. Performing such a study only for scientific purposes would not bring practical results, and ethically it would be controversial. Moreover, the study lasted 3 years, during which the perception of pMRI by neonatologists changed. At the beginning, the majority of children had a repeat MRI examination immediately after birth. As time passed, before the publication of the study results, it was observed that postnatal MRI began to take place later in time because “pMRI results were available”.

Prenatal magnetic resonance imaging was characterized by:

- high agreement of diagnoses of ventriculomegaly,
- relatively high agreement of diagnoses of midline defects,
- relatively high agreement of diagnoses of posterior fossa defects,
- fairly high agreement of diagnoses of neuronal angulation and migration defects and of neuronal proliferation defects.

Prenatal MRI was characterized by high agreement of diagnoses in the case of defects accompanied by defects of the central nervous system, and low in the case of complex defects of the central nervous system.

### 4.2. What Is Known

Defects of the CNS are the fourth-most-frequent presentation of congenital structural malformations. In order of occurrence, the most common are: heart defects, musculoskeletal system defects, and urinary system defects. However, CNS defects are characterized by the highest mortality in the first year of life and are often associated with a delay in mental and psychophysical development. The gold standard in the detection of congenital malformations is ultrasound examination. In accordance with the Regulation of the Minister of Health and the guidelines of the Polish Gynecological Society, during pregnancy, every woman should have at least four ultrasound examinations. In the case of suspicion of a developmental defect, diagnostic testing may be extended to MRI [5,6].

In recent years, two review studies summarizing previous achievements and one meta-analysis have been conducted and the results of a multi-center prospective cohort study, MERIDIAN, have been published [6]. Studies carried out in recent years have clearly established the role of MRI in prenatal diagnosis. The controversies are more related to the magnitude of the effect of pMRI and its indications, when it should be recommended. All experts emphasize that ultrasound and pMRI are complementary tests that enhance each other. pMRI visualizes areas that are poorly visible on ultrasound and vice versa. Both tests are a part of complex prenatal diagnostics. Physicians, the technicians conducting the examination, thanks to previous test results of a given fetus, know to which features they should pay special attention.

In the case of CNS defects, complex defects are often found. The coexistence of several defects has a significant impact on postnatal prognosis. The nature of the defect and the presence or absence of additional abnormalities are important for the child’s psychomotor development. It has been shown that, in the case of mild and moderate ventriculomegaly, fetal defects not detected on ultrasound may coexist. However, the reported inconsistency rate in 2020 is lower than in previous reports. Similarly, in fetuses with isolated anomaly of the corpus callosum diagnosed on antenatal neurosonography, MRI can identify a small proportion of additional anomalies, mainly malformations of cortical development, which are not detected on ultrasound. If ventriculomegaly or corpus callosum anomaly is detected on ultrasound, pMRI should be considered [7,8].

In the literature evaluating the consistency of prenatal and postnatal diagnoses, there are no clear explanations on the course of action in the case of coexistence of defects. The methodologies include assumptions that in such situations the main diagnosis was chosen [9]. This is a very general statement by which it is difficult to trace diagnostic accuracy. In the case of coexistence of agenesis of the corpus callosum and agenesis of the cerebellar vermis, it is not easy, if at all possible, to choose which defect is more important, main or dominant. It is not known whether in the case of agenesis of the corpus callosum, a lack of prenatal recognition of gray matter heterotopia was considered consistent of diagnoses [10]. In this study, in the case of complex defects, a complete consistency in diagnoses was assessed. This means that if there was an inconsistency in even one main diagnosis, the entire diagnosis was considered inconsistent. This approach resulted in a low Cohen’s kappa coefficient κ = 0.21.

Blaicher compared pre and postnatal MRI on a group of thirteen fetuses with CNS defects. In all cases, the diagnoses were consistent [11]. In 2010, Herman-Sucharska et al. examined the consistency of prenatal diagnoses using pMRI with postnatal diagnoses on a group of 73 fetuses (67 fetuses based on clinical examination, 6 fetuses based on autopsies) finding as much as 95.8% consistency. The defects mainly concerned CNS defects [12]. Whitby, in his work, showed that in more than half of the cases, pMRI confirmed the results of ultrasound examination. In 29% of cases, he changed the diagnosis, of which in 11% he excluded the suspected defect confirming normal imaging results of the brain. No false positives were found for pMRI or in cases where ultrasound examination would provide more information [13]. Kul in 2012 showed that MRI provides additional information not only in cases of CNS defects, but also in cases of defects of other systems: digestive, urogenital, and thoracic defects [14].

The assessment of the fetus is always subject to error related to the intrauterine environment. Despite advanced prenatal techniques, obstetricians, pediatricians, and radiologists always stipulate that a full assessment will be possible only after the birth of the child. Nevertheless, researchers are looking for ways to replace postnatal tests with prenatal tests. In 2004, Blaicher postulated that pMRI should replace MRI of the newborn [11]. In 2013, Bekiesińska-Figatowska showed that the uterus can be a “natural incubator”. pMRI was conducted twice during pregnancy on a group of 31 fetuses and the results were compared with each other.

In the majority of cases, the second study was a follow-up after intrauterine myelomeningocele repair. In other cases, a follow-up was necessary due to inconclusive ultrasound results and to monitor the course of the disease. Similarly in 2011, on a small group of fetuses, pMRI and nMRI were compared in imaging brain tumors, showing similar efficacy. Performing MRI examination in a fetus is easier and safer than in a newborn [15,16]. Miller based the qualification for neurosurgery on pMRI results with good outcomes [17].

The comparison of pMRI results with various postnatal studies could be considered as a weakness of the study. Among other authors, only a few compared pMRI with nMRI; the majority, similarly to the conducted study, referred to various postnatal examinations and even to autopsy findings and postmortem imaging studies.

A strength of the study is that pMRI was conducted at a similar time. An innovative idea is to perform pMRI around 35 weeks of pregnancy. Thanks to this, one can better compare to postnatal results.

### 4.3. Future Implications

With the development of technique, diagnostic capabilities, our knowledge about the pathophysiology of defects and genetic and prognostic factors, it is necessary to constantly reevaluate the usefulness, indications, sequence selection, and time period of pMRI.

## 5. Conclusions

pMRI was characterized by low consistency of diagnoses in the case of complex brain defects. The possibility of replacing postnatal studies with pMRI was not supported.

## Figures and Tables

**Table 1:** Statistical analysis in relation to certain CNS defects.

	McNemara’s Test: χ^2^ (p)	Cohen’s Kappa Coefficient: κ (95% CI)		Newborn	
CNS Defect			Fetus	0	1
Ventriculomegaly			0	29	1
	0.25 (0.62)	0.87 (0.74–0.99)	1	3	27
Central line defects			0	29	6
	0 (1)	0.55 (0.34–0.77)	1	7	18
Defects of the posteriori fossa			0	46	2
	0.57 (0.45)	0.6 (0.33–0.86)	1	5	7
Disorders of neural migration and proliferation			0	48	6
	2.29 (0.13)	0.53 (0.23–0.83)	1	1	5
Others			0	41	4
	0.13 (0.72)	0.64 (0.42–0.87)	1	4	11

0—lack of defect, 1—defect present. Please consider each section for separate defects. The lines correspond to prenatal MRI, and vertical intersections specify the number of patients with CNS defects confirmed on postnatal examination and the number of patients without postnatal defects.

## Data Availability

Data available on request from the corresponding author.
